# Respiratory Activity during Exercise: A Feasibility Study on Transition Point Estimation Using Impedance Pneumography

**DOI:** 10.3390/s21186233

**Published:** 2021-09-17

**Authors:** Marcel Młyńczak, Hubert Krysztofiak

**Affiliations:** 1 Institute of Metrology and Biomedical Engineering, Faculty of Mechatronics, Warsaw University of Technology, 02-525 Warsaw, Poland; 2 Department of Applied Physiology, Mossakowski Medical Research Institute, Polish Academy of Sciences, 02-106 Warsaw, Poland; hkrysztofiak@imdik.pan.pl

**Keywords:** exercise, respiratory steady-state, elite athletes, non-invasive respiratory monitoring, gas exchange threshold

## Abstract

The current diagnostic procedures for assessing physiological response to exercise comprise blood lactates measurements, ergospirometry, and electrocardiography. The first is not continuous, the second requires specialized equipment distorting natural breathing, and the last is indirect. Therefore, we decided to perform the feasibility study with impedance pneumography as an alternative technique. We attempted to determine points in respiratory-related signals, acquired during stress test conditions, that suggest a transition similar to the gas exchange threshold. In addition, we analyzed whether or not respiratory activity reaches steady states during graded exercise. Forty-four students (35 females), practicing sports on different levels, performed a graded exercise test until exhaustion on cycloergometer. Eventually, the results from 34 of them were used. The data were acquired with Pneumonitor 2. The signals demonstrated that the steady state phenomenon is not as evident as for heart rate. The results indicated respiratory rate approaches show the transition point at the earliest (more than 6 min before the end of the exercise test on average), and the tidal volume ones at the latest (less than 5 min). A combination gave intermediate findings. The results showed the impedance pneumography appears reasonable for the transition point estimation, but this should be further studied with the reference.

## 1. Introduction

There is constant scientific, clinical, and sports interest in the assessment of the physiological response to exercise. It is often evaluated by a threshold for blood lactate accumulation during progressive exercise [1]. This point, usually referred to as the lactate threshold (LT), sets boundaries between moderate and heavy exercise intensity [1,2,3]. That is why it is a valuable index of physical fitness and a parameter used for exercise programming. It allows predicting exercise performance and controls effects of training in sport and exercise therapy [4,5]. It is used to aid clinical decision-making and stratification for surgical procedures, for example, heart transplantation [6]. Until recently, the term anaerobic threshold was also used [2,7]. However, there is evidence that working muscles are not anaerobic at the LT; thus, the term should be discarded [1].

For direct LT detection, the blood samples for determining the lactate concentration, taken at each step of the graded exercise test, are needed, making this direct method invasive [2,4,5]. Next, mathematical modeling to detect the threshold must be applied, and, considering the non-continuous manner of the sampling, the reliability of the assessment is limited though direct [2,4,5]. The latest paper presented the monitor for such an analysis, which significantly improves the convenience of testing; however, they still do not provide the possibility to record changes continuously [8]. The reproducibility and correlation of LT with sports results were also tested [9].

However, since the invention of respiratory gas exchange measurement instruments, referred to as the ergospirometers, non-invasive methods for detecting a threshold equivalent to the lactate threshold (the gas exchange threshold) were introduced that rely on respiratory gas exchange measurements [7,10]. These methods are advantageous because measurements are not only non-invasive, but sampling is almost continuous, on a breath-by-breath basis.

The phenomenon behind the gas exchange threshold is related to the buffering of metabolic acidosis resulting from hydrogen ions accumulation as a consequence of hydrolysis of ATP generated by glycolysis. The buffering of metabolic acidosis by blood bicarbonate engenders CO${}_{2}$ release, in addition to the metabolic CO${}_{2}$ output linked to oxygen uptake [11]. That excesses CO${}_{2}$ results in enhanced respiratory activity. The gas exchange threshold may be identified by analyzing the slope of the regression line of carbon dioxide output against the oxygen uptake or the regression line of minute ventilation against workload [7,10].

The ergospirometers for gas exchange threshold detection are widely used, and the methodology is established [12]. However, the equipment is expensive [13], the testing procedure is quite complicated (particularly from the subject’s perspective), needing regular calibration, and the oro-nasal mask used during the test may introduce discomfort and breathing disturbances in the examined subject. Furthermore, not a lot of new ideas in this regard have appeared recently, but noteworthy is the structure of the mask divided into the nasal and oral flow [14].

Hence, the constant search for another method for threshold detection, to make the measurements even more non-invasive: a method that would make the procedure simple, easily accessible, and more convenient for the subject, especially for patients with respiratory or cardiological limitations; a method that would be reliable, as well, of course. Several methods that utilize electromyography recordings or heart rate variability analysis based on electrocardiography signals have been presented [15,16,17,18,19]. However, those approaches seems very indirect; therefore, they should be treated rather as illustratory.

Returning to the mechanical monitoring of ventilation, but less obtrusive and more comfortable (lack of the need for the face mask, which frees the breathing space during exercise), it seems impedance pneumography can be such a simple, convenient, and reliable method. It is based on measuring transthoracic impedance changes, resulting from successive inspirations and expirations, through ECG-like electrodes placed on the subject’s chest. The previous research showed that the tetrapolar approach with the electrodes explicitly placed on arms and under the armpits provides linearly consistent results with the volume of air being inhaled and exhales [20,21]. Therefore, we recognized that method to be used even without calibration to measure relative changes, not absolute values, with a simple assumption that a change of the impedance amplitude twice means a corresponding change in the air volume twice [22,23].

Therefore, we decided to perform the feasibility study on the possibility to determine points in respiratory-related impedance pneumography signals, acquired during stress test conditions, that suggest a transition similar to the gas exchange threshold? Secondary objective, which should be analyzed before the main aim, is to answer: does respiratory activity reach steady states during graded exercise?

## 2. Materials and Methods

### 2.1. Participants and Protocol

Forty-four students (35 females) practicing sport to varying degrees (from a very amateur level to an almost competitive one) volunteered to participate in this study and perform a graded exercise test. All the participants were healthy, and none of them was taking any medication. During the initial analysis, we decided to remove four recordings (due to bad signal quality preventing the detection of any respiratory onsets and phases, probably due to poor preparation of space for electrodes attachment), then the other three (due to the failure to maintain the protocol by the subject), and, finally, the other three (due to too short registration—interrupted before three entire segments are finished). Eventually, there were 25 females and 9 males included in this feasibility evaluation. Their characteristics are provided in Table 1.

The test started with a workload of 40 W, and the workload was increased by 40 W every 3 min until volitional exhaustion.

The study was carried out at the National Center for Sports Medicine in Warsaw. The Ethics Committee of Warsaw Medical University approved it (permission AKBE/74/17). All participants provided written informed consent and were informed about the general aim of the measurements, though not about the importance of breathing activity because this did not affect their registrations.

### 2.2. Devices and Measurements

The measurements were performed in a diagnostic room designated for exercise tests and other sport-related examinations. We used an Ergoselect 100 ergometer (Ergoline, Bitz, Germany) and BTL-08 SD ECG (BTL Industries Ltd., Herfordshire, UK) to coordinate the protocol, record point-wise heart rate value at the end of each analyzed stage, and control the subjects, particularly during the last phase of the exercise.

We also used Pneumonitor 2 as a device that uses impedance pneumography technique to collect respiratory-related changes in the electrical impedance of the thorax in relation to the changes in the amount of air in lungs. The device uses the tetrapolar method in which application current is delivered through a pair of electrodes. The application part of the sensor comprises a controllable voltage generator (fixing the shape and frequency of the signal), the voltage-to-current converter (establishing the amplitude), and impedance transformation module. The application circuit enables to get the current that has constant amplitude regardless of load changes. The voltage, in turn, is measured by other two electrodes (what enables obtaining better distribution of current density and neglecting the negative impact of the skin-electrode connection impedance). The receiving system consists of instrumental amplifier, amplitude demodulator in the form of a diode peak detector, and signal conditioning circuit composed of matching amplifiers and analog filters. Then, the signal is digitally converted and further processed by the STM32 microcontroller. The more extended technical description of the device and the method (e.g., electrode configurations used) was provided in Reference [24].

The specified electrode configuration, which allows to get the highest linearity of the relation between measured impedance and tidal volume, was chosen according to the study of Seppa et al. [20]. The configuration consists of placing receiving (voltage) electrodes on the mid-axillary line at about the 5th-rib level, and positioning application electrodes on the same level on the insides of the arms.

The Pneumonitor 2 enables to record the data with the sampling frequency of 250 Hz. In both ECG and impedance pneumography, standard Holter-type, disposable ECG electrodes were used. They are connected to the device using shielded medical cables.

We assumed that the accuracy and stability of the impedance pneumography signals were similar to the presented in References [21,25].

**Figure 1:**
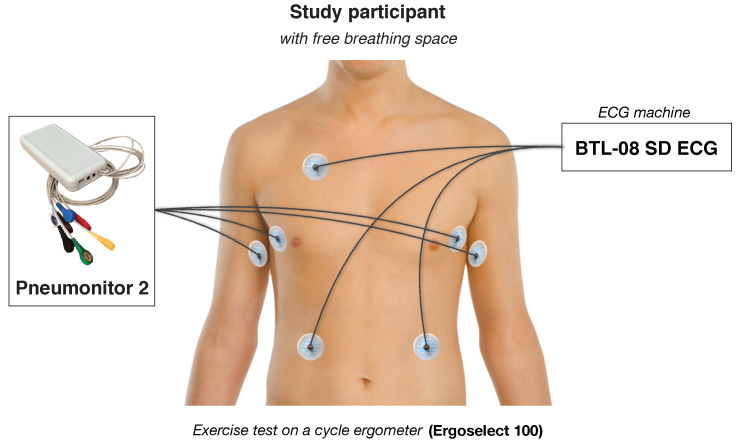
The scheme of the configuration of measurement equipment and devices.

A schematic configuration of measurement equipment and devices attached on subject, illustratively describing how the data were collected, is presented in Figure 1.

### 2.3. Ventilatory Response during Graded Exercise

For each subject, we recorded or estimated several parameters:

- Workload and workload indexed to body mass;
- Heart rate (HR, recorded during last minute of a segment);
- Respiratory rate: determined as averages at 30 s intervals with 50% overlap, each value is calculated from the distance between two detected respiratory onsets;
- Tidal volume (TV): the initial one denoted as 1, then determined as averages at 30 s intervals with 50% overlap;
- Complex respiratory parameter constructed as multiplication of corresponding respiratory rate and relative tidal volume (here and after VENT); a surrogate of such an approach was presented in Reference [26].

To analyze the possible existence of respiratory steady states during graded exercise, we have set an objective criterion that the steady state phenomenon is present when the variation of the results in the last minute of the segment is significantly smaller than for the previous 2 min. To assess it statistically, a two-sample F-test for equal variances was applied on respiratory rate, tidal volume, and VENT data.

On the other hand, we believe that the possible steady state phenomenon can also be expressed and analyzed using a graph similar to the Poincare plot for RR intervals [27], but with respiratory rate points or with VENT values (such as a combined relative rate and depth of breathing variability analysis). The similar approach was presented in a paper by Welch et al. for a different purpose [28]. As for the Poincare analysis, SD1 (short-term) and SD2 (long-term) parameters can be calculated. We hypothesize higher short-term respiratory stabilization will be connected mainly with low SD1 values. However, as this is a novel approach, there is no reference to establish which SD1 values can be low enough; hence, this was estimated only for illustration.

### 2.4. Transition Point

As this is a feasibility study of the use of impedance pneumography in the stress test conditions, we decided to skip analyzing the blood lactate concentration or respiratory gases. From the perspective of a gas exchange threshold estimation, this can treated as a main limitation of the study (mentioned in the Discussion).

**Figure 2:**
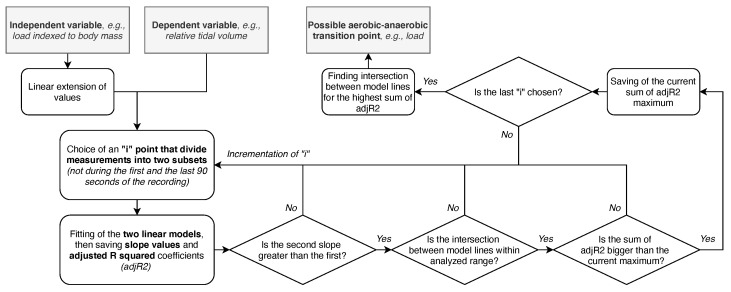
The idea of how the transition point suggesting gas exchange threshold can be searched.

However, we still browsed for the transition point that may suggest a gas exchange threshold to evaluate if anything can be found. We established the procedure to find the point for which there is the optimum of the sum of adjusted R squared coefficients of two linear models fitted for all points before and after such hypothetical “inflection” point. Workload and HR were extended linearly from the beginning to the end of the recording to enable models fittings, i.e., after the operation, the workload within a single study segment remains the same for the middle of this segment. It was also arbitrarily assumed that the point could not be during the first (too early) and the last (too late, if the end is at the state of exhaustion) 90 s of the recording. Furthermore, the presented method is sensible when the second slope is greater than the first, and the intersection between model lines is within the analyzed range. The scheme of the search algorithm is presented in Figure 2.

The search was performed on six relations between:

- VENT and workload indexed to body mass,
- Respiratory rate and workload indexed to body mass,
- Relative tidal volume differences and workload indexed to body mass,
- VENT and HR,
- Respiratory rate and HR,
- Relative tidal volume differences and HR.

In addition, general averages that took into account values estimated using the former three and the latter three approaches were analyzed. Analysis of variance was used to evaluate the impact of the groups, divided according to:

- sex,
- physical capacity (arbitrary two groups: those who completed more than five stages and those who completed five full stages or less), and
- initial heart rate (measured just before the start of the test; divided into two groups: below or above 90 BPM—probably anticipatory HR rise was already present at the beginning, and there would be a smaller space for further growth),

on found transition points.

All signal processing, data analysis and statistics were carried out using MATLAB 2020b software (Mathworks, Natick, MA, USA). The data analysis was elaborated by the first author, then reviewed by another one, and, finally, performed by the first one.

## 3. Results

During their measurements, the subjects performed the stress test and finished full:

- 3 steps (120 Watts): 6 females,
- 4 steps (160 Watts): 10 females,
- 5 steps (200 Watts): 7 females,
- 6 steps (240 Watts): 1 female and 2 males,
- 7 steps (280 Watts): 1 female and 4 males,
- 8 steps (320 Watts): 3 males.

### 3.1. Ventilatory Response during Graded Exercise

**Figure 3:**
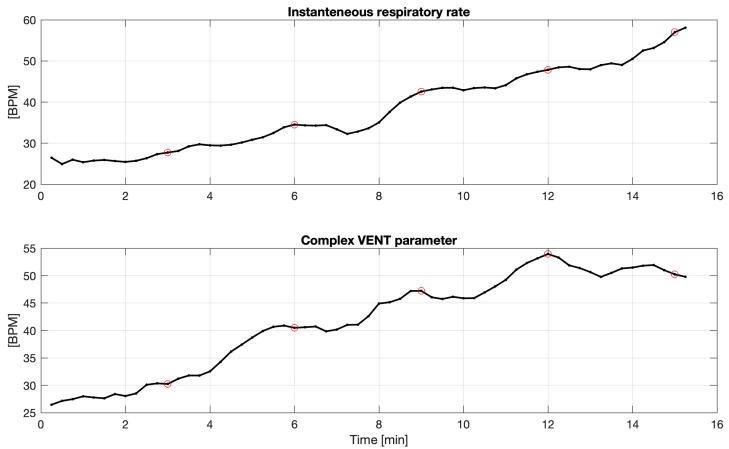
Sample courses of respiratory rate and complex VENT parameter for IP signal acquired for the 34th subject.

For all 34 subjects, we observed no statistically significant difference (between the first two minutes in the segment comparing to the last third minute) for respiratory rates (assessed by the variance tests, described in Section 2.3). Only for two subjects (almost 6%) was there a significant difference in tidal volume (for the other two, *p*-values between 0.05 and 0.08 were also observed). For one of them, sample smoothed courses of respiratory rate and complex VENT parameter are presented in Figure 3.

**Figure 4:**
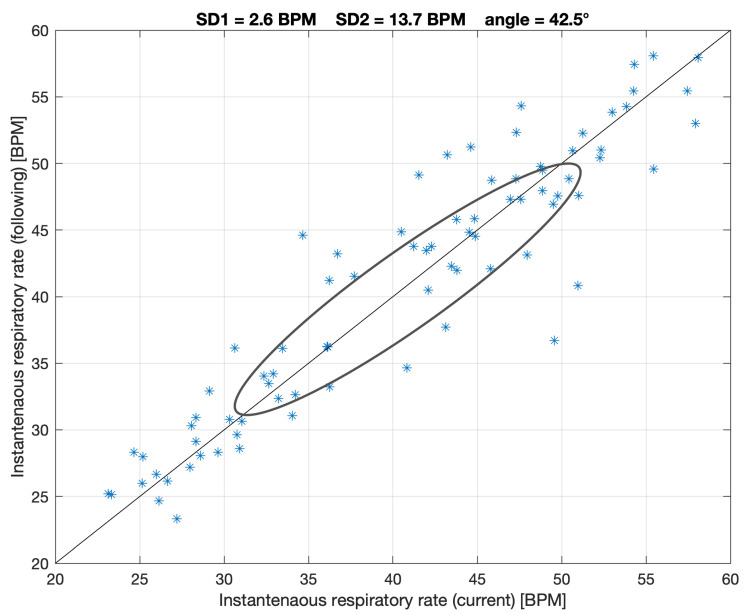
Sample figure presenting respiratory-related Poincare-plot equivalent for the respiratory rate case, for the 34th subject. Angle means the tilt of the ellipse fitted and is presented illustratively.

The analysis of respiratory-related Poincare plot performed for respiratory rate and VENT parameters showed very similar SD1s, for the former, and more diversified SD1s, for the latter, respectively. Table 2 gathers both short-term and long-term variabilities (as mean ± SD), along with their average ratios. Sample figure presenting respiratory-related Poincare-plot is shown in Figure 4. Higher respiratory rates were gathered in the final phase of the exercise test for high workloads.

### 3.2. Transition Point

As described in Section 2.4, 8 various “inflection points” can be found for:

- IP1: the relation of VENT and workload indexed to body mass,
- IP2: the relation of Respiratory rate and workload indexed to body mass,
- IP3: the relation of Relative tidal volume differences and workload indexed to body mass,
- IP4: the average of the previous three approaches (workload-indexed-to-body-mass-related),
- IP5: the relation of VENT and HR,
- IP6: the relation of Respiratory rate and HR,
- IP7: the relation of Relative tidal volume differences and HR,
- IP8: the average of the previous three approaches (HR-related).

For the first group (IP1–IP3), after their estimation, all were converted back to workload units. The calculation for IP5–IP7 did not need to be modified.

Table 3 gathers results of ANOVA carried out on estimated points versus sex, physical capacity, and initial HR factors, along with basic statistics of the sum of adjusted $R^{2}$ calculated for the optimum case and the time interval from found “inflection point” and the end of stress test. As the independent variable form (one value for the interval) and processing are the same for both workload and heart rate, sums of adjusted $R^{2}$ and time intervals from IP and end of the stress test are similar (as indicated in the table).

For the relation of VENT parameter versus workload indexed to body mass and heart rate, in both cases, estimated transition points differed for physical capacity factor and were insignificantly different for sex and initial heart rate factors. Average sums of adjusted $R^{2}$ were above 1.5. This shows the connection of respiratory rate and relative tidal volume parameter is sensible from the search of the transition point perspective.

Considered factors appeared not differentiated for respiratory rate. In addition, the sums of adjusted $R^{2}$ were the lowest for this case, which means the expected relations occurred in the slightest degree for the temporal-based respiratory parameter. In turn, amplitude-based relative tidal volume was different for sex factor (significantly for the relation with workload indexed to body mass and the trend found for the relation with heart rate).

The results also showed that the approaches, which use respiratory rate parameter, find the transition point at the earliest (6.17 min before the end of a stress test on average), and the approaches, which use relative tidal volume parameter at the latest (4.92 min before the end). VENT-related approaches and IP4 and IP8 approaches gave intermediate results (for IP1, it is about 5 min 48 s, and, for IP4, about 5 min 38 s).

**Figure 5:**
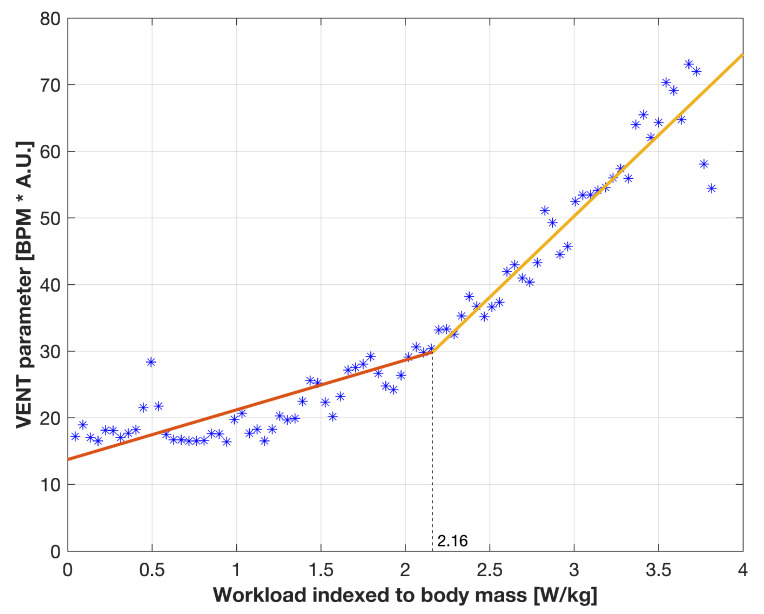
The sample relation of VENT parameter and workload indexed to body mass for the 19th subject (see details in the text) with the linear models providing the estimated transition point. The left one can described by: VENT = 7.46 × WLiBM + 13.73; the right one by: VENT = 24.33 × WLiBM − 22.70, where WLiBM is workload indexed to body mass. The independent variable is linearly distributed, as described in Section 2.4.

Figure 5 presents the sample relation of VENT and workload indexed to body mass for the 19th subject (male, stopped the test at 280 W with declared Borg scale of 18) with two models fitted for the data before and after found transition point. After converting to workload units, the transition point of 2.16 W/kg was estimated at 178.36 W (the heart rate at the end of this segment was 154 BPM).

Furthermore, Table 4 gathers individual results of all participants.

## 4. Discussion

In this article, we attempted to use impedance pneumography during stress test conditions to answer the questions of how the respiratory activity changes during exercise. The impedance pneumography measurement appears very comfortable from the subject’s perspective; it is easy to use, and it enables to record the signal related to tidal volume (if the specific electrode configuration is chosen) with very high sampling rate (even higher than necessary). The circuit for respiratory-related impedance measurements can be also combined with ECG one to assess cardio-respiratory fitness (also using the same wires [24]).

The first issue concerned specifically the course of ventilatory activity changes during graded exercise in terms of the occurrence of steady state at each stage of submaximal exercise. The test’s inspiration came from the observation that, during graded exercise tests, at each stage (usually lasting 3 min), a steady state for cardiological parameters is achieved [29]. Accordingly, one can notice a time-dependent decrease of RR-intervals variability and complexity, while exercise intensity increases.

The obtained result for breathing demonstrated that the steady state phenomenon is not as evident as for heart rate. It is coherent with our expectations and further allowing the assumption of using the impedance-pneumography-related signals for the estimation of transition point that may be similar to a gas exchange threshold.

On the other hand, we can speculate that the stabilization of breathing mechanics may appear, though for longer single stages and only before reaching the actual gas exchange threshold. Probably, before stabilization is achieved, the next stage starts—the workload increases. This requires further studies with a dedicated prospective protocol. On the other hand, very similar short-term variabilities found for respiratory rates may indicate a different response mechanism, which is more temporary, not directly connected with the scheme of load changes during the test. In turn, tidal volume data do not support such an interpretation. Presumably, the driver for tidal volume was not controlled.

Another issue was the search of the points in the breathing signals that suggest a transition similar to gas exchange threshold. The estimation of the ventilatory thresholds during exercise by measuring only the respiratory rate has already been attempted and presented in a number of previous investigations [30,31,32,33]. However, as respiratory rate and tidal volume are regulated differently during exercise, including an incremental one [34,35], we decided to test the impedance pneumography technique for this purpose with the access to the signal from which both respiratory rate and tidal volume curves may be extracted. It is an indirect method, and this can be considered a drawback [36]. However, it allows operating without any breathing space distortion or any collecting blood samples at the end of each study segment. It can show the prospect of a more straightforward test approach [37].

Furthermore, the search for a single point is used only as a simplification for a practical application. During a graded exercise, one can sometimes identify even two breakpoints, the so-called first and second ventilatory thresholds (the latter is also known as respiratory compensation point). We cannot unequivocally evaluate if the estimated inflection points corresponds to the gas exchange threshold or to the respiratory compensation point. Therefore, we decided not to choose the “optimal” approach at this stage. We focused on assessing the possibilities, not on performing a comparative analysis. In the study, the estimated transition point was by the average between almost 5 to over 6 min before the volitional end of the test, what can be interpreted as the range of transition domain. Considering the group comprises mainly amateurs, it seems sensible; in the study of Scherr et al. [38], the average individual anaerobic threshold appeared at the level of 13.6 Borg scale (12.9±2.7 in our study).

In addition, mathematically, when analyzing a single subject and single stress test results, there are no differences in the final estimation with or without considering indexing workload to body mass. We, however, decided to focus on the former approach, as it would be universal; the point can be estimated for the same conditions and the same patient, but with different body mass; or it enables to compare various subjects, mainly two genders significantly differing with the body mass.

### 4.1. Study Limitations

This work was primarily to show the use of impedance pneumography method in the stress test conditions, and specific analytical methods to try solving a physiological problem. It was not our goal to establish any overall results or conclusions for the entire population.

As this is a feasibility evaluation, it has several limitations. Firstly, there is no reference, which can be used to compare and validate the estimated transition points. However, the reference itself appears controversial, as it is hard to indicate a single point, which fulfills all criteria to become the threshold. There are several drawbacks mentioned in the literature [1,39,40].

Secondly, the study is performed on a small, uneven, and age-homogeneous group comprising only healthy subjects. As the inference may concern elite athletes, the participants could even be too amateur. We decided to cut off the registrations for those subjects who did not finish 3 steps. In addition, there are 16 (47%) subjects that ended the test after 3 to 4 steps (the registration then lasting about 9 or 12 min, respectively). It appears short and diversified across the entire group; however, the assessment is limited to the distance from the transition point to the end of the registration, as well as the quality of the two fitted linear models. The presented methodology can be reasonably performed on such short data.

Next, even for changes of an exponential nature, it is difficult to indicate one inflection point. Our approach to optimize two linear models was only one of the possible ones.

Furthermore, ANOVA results are only illustrative due to small N, and, as the factors are not general, mainly, initial heart rate is difficult to interpret. It may indicate imprecise control over participants’ preparation for the study.

It is also more common to use an exercise duration of 6 min to test breathing steady states. For the final conclusion on the existence, or not, of the steady state for breathing, the results should be compared to reference values, such as heart rate or blood lactate concentration.

It appears that the time immediately after exercise is also crucial to evaluate how fast and how efficiently the recovery and the rest state is achieved [41]. However, it was not further analyzed.

### 4.2. Novelty

We believe the value of the paper lies in the application of the method in the specific conditions and in the analysis of data to present the possible way of solving the physiological problem of finding a point similar to gas exchange threshold (this still should be further studied with the reference). In addition, despite the mentioned limitations, to our knowledge, the paper describes for the first time:

- the use of VENT parameter, which combines both tidal volume and respiratory rate, in the specific application of the dynamic respiratory mechanics analysis, and
- various analytical approaches based on impedance pneumography signals to find an estimation of a transition point (that might be further studied to be similar to gas exchange threshold).

### 4.3. Further Research Perspective

The presented study shows the possibility to use impedance pneumography in more dynamic conditions as usually presented (eventually outside the laboratory). It was tested before, e.g., in Reference [42]; however, the method has not been significantly developed for the sports medicine purpose yet. This can create a space for further research.

The sensor can be improved by replacing the device fixed to the pants strap, with electrodes connected via cables by a patch positioned at the level of sternum. On the other hand, the Ag/AgCl electrodes could be replaced by more wearable ones, i.e., made of graphene layers.

Moreover, as the mechanical activity of breathing is relatively rare analyzed together with cardiac activity, impedance pneumography can be chosen to quantitatively measure cardiorespiratory characteristics during a stress test.

Other interesting issues are:

- researching a larger group, with the participation of elite athletes, with the assessment of blood lactate concentration and/or the ergospirometric examination as a reference to comparison with impedance-pneumography-based estimations,
- further assessment of respiratory response during the graded exercise test in terms of possible long-term steady state occurrence, but with modified protocol comprising longer stages of constant workloads,
- evaluation of age-, sex-related, and health-state-related differences in cardiorespiratory characteristics, similar those to presented in Reference [43],
- performance of long-lasting study considering training period and/or the course of a season and associations between cardiorespiratory fitness and actual workload (not only forced by the exercise stress test), the inspiration for which comes from Reference [44],
- supplementing the analysis to assess the flow-volume curve “movement” during the test, depending on fatigue levels (also going towards recovery state after exercise test).

## 5. Conclusions

Impedance pneumography is the method, which can be considered to be used for stress test conditions. It provides continuous data, does not distort natural breathing, does not affect the subject’s comfort, and enables recording of both relative tidal volumes single lead ECG signal, using the same configuration of electrodes.

The obtained data from the graded test demonstrated that the steady state phenomenon is not as evident as in the case of heart rate. This facilitates testing the concept of using mechanical respiratory data for the estimation of a transition point, which is similar to gas exchange threshold.

These estimations performed with the described algorithm indicated that one can find inflection points in parameters derived from impedance pneumography signals. The respiratory-rate-related approaches showed the point at the earliest (more than 6 min before the end of the exercise test on average), and the tidal-volume-related ones at the latest (less than 5 min before the end of the test). Others gave intermediate estimates (around 5–6 min before the end of the test).

As it was a feasibility study, all aspects should be still further studied with the physiological reference.

## Figures and Tables

**Table 1:** Demographic characteristics of the study group; values presented as mean ± standard deviation.

	Female	Male
N	25	9
Age [y]	24 ± 1	25 ± 1
Height [cm]	167.8 ± 4.5	182.9 ± 4.0
Weight [kg]	60.0 ± 7.8	77.9 ± 5.6

**Table 2:** The statistics of respiratory-related Poincare-plot equivalent characteristics described by SD1, short-term variability, SD2, long-term variability, and their ratio, for analyses performed for respiratory rate and VENT parameter cases; BPM stands for breaths per minute.

Case	SD1	SD2	Avg. SD1/SD2
Respiratory rate case [BPM]	1.47 ± 0.06	8.17 ± 1.04	0.182
VENT parameter case [a.u.]	4.51 ± 0.65	34.42 ± 3.67	0.134

**Table 3:** The results of ANOVA analyses (only *p*-values lower than 0.1 were provided; otherwise, they are labeled as “Ns”.) and statistics of the sum of adjusted $R^{2}$ calculated for the optimum case and the time interval from found “inflection point” to the end of stress test. “*” means *p*-values between 0.05 and 0.01; “**” means *p*-values between 0.01 and 0.001.

Approach to Estimate the Transition Point	ANOVA *p*-Value for Sex Factor	ANOVA *p*-Value for Physical Capacity Factor	ANOVA *p*-Value for Initial HR Factor	Mean ± SD Sum of Adjusted $R^{2}$ for the Optimum Case (Max. 2)	Mean ± SD Time from IP to the End of Stress Test [minutes]
IP1	Ns.	0.001 **	Ns.	1.56 ± 0.21	5.82 ± 3.43
IP2	Ns.	Ns.	Ns.	1.07 ± 0.38	6.17 ± 3.96
IP3	0.002 **	Ns.	Ns.	1.33 ± 0.32	4.92 ± 3.63
IP4	0.006 **	0.005 **	Ns.	—	5.63 ± 2.22
IP5	**0.07**	0.026 *	Ns.	As for IP1	
IP6	Ns.	Ns.	Ns.	As for IP2	
IP7	**0.07**	Ns.	Ns.	As for IP3	
IP8	Ns.	Ns.	Ns.	—	As for IP4

**Table 4:** The individual results of all participants including (1) workload at the transition estimated as an average from all approaches (IP1–IP8 ); (2) average interval from the transition to the end of stress test (IP1–IP3); (3) and (4) SD1 and SD2 from the Poincare respirate-rate-related analysis; all linked to demographical data (sex and BMI), the number of completed steps (fitness level equivalent), and final HR (at the end of the stress test).

ID	Sex	BMI	Completed Steps	Final HR	Workload at the Transition [Watts] (1)	Interval to the Test End [min] (2)	SD1 [BPM] (3)	SD2 [BPM] (4)
1	F	23.5	6	189	170.5	5.1	1.57	8.12
2	F	22.2	4	189	125.6	4.7	1.43	6.26
3	F	21.8	4	173	109.6	4.7	1.36	6.62
4	F	18.9	4	166	79.0	4.7	1.28	6.70
5	F	18.6	4	176	102.2	3.8	1.35	6.30
6	F	20.5	5	189	160.3	4.3	1.31	7.48
7	M	23.6	6	177	117.1	9.3	1.43	7.30
8	M	24.2	6	175	172.0	7.4	1.44	7.16
9	M	21.2	7	180	159.2	6.1	1.50	7.18
10	F	18.3	3	174	93.5	4.4	1.49	7.05
11	F	20.8	5	181	111.3	5.3	1.49	7.75
12	F	20.4	5	178	79.3	8.2	1.45	7.81
13	F	20.7	4	179	88.4	5.3	1.44	7.80
14	F	23.3	5	184	74.3	7.8	1.45	7.64
15	F	20.9	3	170	90.6	5.5	1.46	7.50
16	F	19.4	7	190	204.7	5.3	1.43	7.32
17	F	22.9	4	195	63.0	6.5	1.43	7.22
18	M	25.9	8	193	142.5	9.3	1.47	8.32
19	M	22.6	7	182	152.6	7.3	1.48	8.91
20	F	20.4	5	183	126.0	6.7	1.49	8.91
21	F	21.7	5	198	112.2	6.1	1.48	8.81
22	M	20.3	7	191	148.2	8.8	1.53	8.99
23	F	22.9	4	200	154.9	2.2	1.52	8.92
24	M	22.2	8	190	177.1	8.9	1.52	9.35
25	F	23.7	4	160	124.9	3.5	1.51	9.36
26	F	25.6	4	164	116.4	2.8	1.51	9.30
27	F	22.1	4	180	93.3	3.8	1.51	9.21
28	F	16.7	3	140	90.5	3.0	1.52	9.13
29	F	18.1	3	184	76.9	4.4	1.51	9.04
30	F	21.0	3	145	103.6	2.4	1.51	8.97
31	M	24.8	8	195	139.2	11.0	1.51	9.11
32	F	30.5	5	195	120.6	3.8	1.50	9.02
33	F	18.4	3	156	89.8	3.1	1.50	8.96
34	M	25.1	7	205	192.8	6.3	1.56	10.23

## Data Availability

Data and materials used in this study are available upon reasonable request to the corresponding author and under a collaboration agreement.
